# Axillary water hammer pulse in severe aortic regurgitation

**DOI:** 10.1093/ehjcr/ytag074

**Published:** 2026-02-06

**Authors:** Minel Soroa, José Rozado

**Affiliations:** Icahn School of Medicine at Mount Sinai, Mount Sinai Fuster Heart Hospital, 1 Gustave L. Levy Place, NewYork, NY 10029-6574, USA; Cardiology Department, Hospital Universitario Central de Asturias, Avenida de Roma s/n, Oviedo, Asturias 33011, Spain

**Keywords:** Aortic regurgitation, Water hammer pulse, Watson's pulse, Physical examination

## Case report

A 78-year-old man presented to the Emergency Department with orthopnea and dyspnoea at rest, following a long-standing history of progressive shortness of breath. On physical examination, he had signs of congestive heart failure, a wide pulse pressure (144/43 mmHg), lateral displacement of the apical impulse, and a high-pitched decrescendo early-diastolic murmur suggestive of significant aortic regurgitation. A striking, regular peripheral arterial pulse was noted, particularly in the axillary arteries (Supplementary material online, *Video SA*), demonstrating a rapid upstroke and sudden collapse. This visible water hammer pulse (also known as Watson’s pulse) is characteristic of significant aortic regurgitation.^1,2^ It occurs due to an increased stroke volume with abrupt distension of large arteries, followed by exaggerated diastolic collapse as blood regurgitates from the aorta into the left ventricle. Echocardiography confirmed severe aortic regurgitation, along with significant left ventricular dilation and systolic dysfunction.

**Figure:**
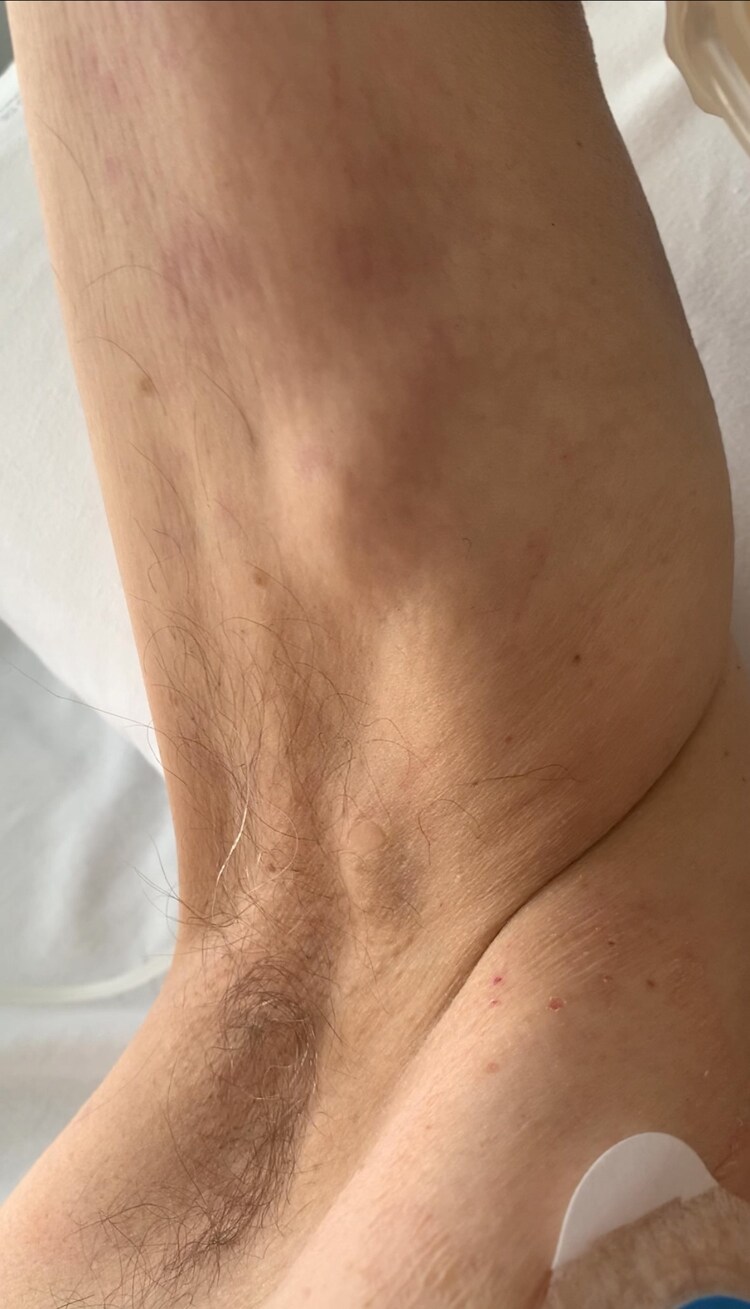


## Supplementary Material

ytag074_Supplementary_Data

## Data Availability

All relevant data are included in the manuscript. Additional deidentified imaging or Supplementary material is available from the corresponding author upon reasonable request.

## Supplementary material

**Consent:** The patient provided informed consent for the collection and use of the video patient in accordance with COPE best practice guidelines. No identifying information is included in the images.
